# Knowledge about age-related eye diseases in the general population in Germany

**DOI:** 10.1186/s12889-024-17889-0

**Published:** 2024-02-08

**Authors:** Jan Henrik Terheyden, David J. Fink, Karl Mercieca, Maximilian W. M. Wintergerst, Frank G. Holz, Robert P. Finger

**Affiliations:** 1 Department of Ophthalmology, University Hospital Bonn, Venusberg-Campus 1, 53127 Bonn, NRW Germany; 2 Department of Ophthalmology, University Hospital Mannheim & Medical Faculty Mannheim, University of Heidelberg, Mannheim, Germany

**Keywords:** Health literacy, Age-related macular degeneration, Glaucoma, Cataract, Diabetic eye disease

## Abstract

**Background:**

With a rising prevalence of age-related eye diseases, prevention and early diagnosis of these conditions are key goals of public eye health. Disease-related knowledge in the general public supports these goals but there is little data available. Thus, we have assessed knowledge of cataract, glaucoma, age-related macular degeneration (AMD) and diabetic eye disease in the German adult general population in a cross-sectional study and identified target groups for health education interventions.

**Methods:**

Knowledge assessment content was identified based on a literature review, expert input, and a list of items was generated after a qualitative selection process. The resulting 16-item instrument (4 items per condition) was administered to 1,008 participants from a survey panel, demographically representative of the adult German population. Test properties were evaluated based on a Rasch model and multiple correspondence analysis (MCA). Binary-logistic regression analysis was performed to investigate associations with age, sex, education level, employment status, marital status, income, reported health status, visual difficulties, and recent general practitioner (GP) and ophthalmologist consultations.

**Results:**

Replies were correct for a median of 9 out of 16 (range 2 – 16) items, which differed between conditions (*p* < 0.0001). Most responses were correct for cataract items (median: 3 / 4) and least were correct for AMD items (median: 2 / 4). 27%, 9%, 1% and 19% of respondents replied correctly to all cataract, glaucoma, AMD and diabetic eye disease-related items, respectively. Rasch analysis suggested an adequate targeting of items and in MCA, no evidence of multidimensionality was present. Older age, being retired, decreased general health and recent GP or ophthalmology consultations were significantly associated with more knowledge about common eye conditions (*p* ≤ 0.005). GP or ophthalmology consultations remained significant in a multivariable model (*p* ≤ 0.011).

**Conclusions:**

Knowledge gaps regarding eye health are considerable in the German general population and should therefore be addressed in educational interventions targeting the public. Special attention when designing such campaigns needs to be paid to infrequent users of the healthcare system. Knowledge of AMD seems to be poorer compared to other eye conditions.

**Supplementary Information:**

The online version contains supplementary material available at 10.1186/s12889-024-17889-0.

## Background

Social determinants of health (SDOH) are major contributors to real-world health outcomes [1]. Health literacy is one of the key components of SDOH, covering aspects of disease understanding and health promotion that exceeds one’s educational background [2–4]. While the definition of health literacy has evolved over the last decades, knowledge about health conditions and their risk factors, prevention strategies, and treatments is well-conceptualized and thus measurable for research, clinical and public health applications. Knowledge about health conditions reflects the most profound level of health literacy (i.e., functional health literacy) [5], and is a major contributor to disparities in health outcomes, including eye health [6, 7].

Patients with chronic eye conditions who are health literate or knowledgeable about eye health have better vision outcomes and are more closely integrated into the healthcare system than those with lower knowledge and health literacy levels [8–12]. Furthermore, the prevalence of age-related eye diseases such as cataract, age-related macular degeneration (AMD), glaucoma and diabetic eye disease rises, and these conditions constitute main causes of vision impairment and blindness worldwide [13, 14]. Therefore, improving the level of knowledge about age-related eye conditions in the general population is an important goal of public eye health.

Despite health-related knowledge having a major positive impact on eye health, very little data on knowledge about age-related eye diseases are available to the general public [15–26]. Better understanding levels of eye health-related knowledge and its determinants in the general population could help target educational interventions, improve health literacy and thus improve eye health outcomes and reduce vision loss. Against this background, we have investigated the level of knowledge of common age-related eye conditions which may lead to vision loss, and of its determinants in a demographically representative sample of the adult German population, by surveying volunteers from an online panel.

## Methods

### Participants and survey design

Survey participants were recruited from the adult German general population to assess their knowledge about cataract, glaucoma, AMD and diabetic eye disease regarding risk factors, prevention strategies, and treatments, using an online recruiting platform (SurveyEngine, Berlin, Germany). Individuals from the general public can register on this platform for participating in surveys and are compensated according to agreements between the participants and the service provider. Eligibility criteria were age ≥ 18 years, German language comprehension, residence in Germany, and internet access. No participants were recruited from other sources than the recruiting platform.

The target size of the surveyed sample was 1,000 participants, considering a population size > 10,000 individuals (adult Germans) and a ± 3% margin of error [27]. The age and sex of participants reflected publicly available data from the German national micro-census 2021 in adults [28], which was implemented in the targeting algorithm of the service provider. Our survey included data on sociodemographic characteristics (age, sex, marital status, level of education, employment, living situation) as well as items on participants’ overall and ocular health (including the EQ-5D-5L questionnaire [29]), use of the healthcare system (number of general practitioner and ophthalmologist consultations over the past 12 months), and sixteen items on knowledge about four common eye diseases (cataract, glaucoma, AMD, diabetic eye disease).

To obtain the knowledge items, a list of contents of existing knowledge assessments instruments on the mentioned ocular conditions targeted at the general population was compiled based on a review of the literature, using the literature search tools PubMed and Google Scholar. Content of existing, published instruments was reviewed by two of the authors (JHT, RPF) and a list of draft items was produced. Four items per disease category were then selected, considering knowledge domains with a clinical or public health relevance. Each item contained a statement about a risk factor, prevention strategy, treatment or public misconception, respectively. Before the instrument was used in the quantitative part of the study, three debriefings with members from the general public were performed to ensure the comprehensibility of the items and response options. We asked participants to rate all statements as true or false (forced response) from their knowledge without support from third parties and with no additional information. Data collection took place in September 2022. Ethics approval was obtained from the institutional ethics committee at the University Hospital Bonn, Germany (approval ID 255/22).

### Statistical analysis

The distribution of variables was graphically inspected and described using the mean and standard deviation (SD), absolute numbers and percentages. In the first part of statistical analysis, we investigated the internal consistency and dimensionality of the 16 knowledge items, using a dichotomous Rasch model, i.e. a one-parameter item response model, under the assumption that the individual items (questions) share a common latent trait [30]. Rasch models describe the probability of a correct response to a questionnaire item as a function of test taker ability and test item properties. We then used the models to assess person reliability, person separation index, item fit, and differential item functioning (DIF) by sex, including female and male participants. DIF was evaluated using contrasts and Rasch-Welch probabilities. Contrasts ≥ 0.64 logits were interpreted as potential DIF and contrasts ≥ 1.0 were interpreted as relevant DIF [31]. Multiple correspondence analysis (MCA) based on a correspondence analysis of the Burt matrix was conducted to further assess knowledge dimensions across disease domains. MCA is a dimension reduction technique similar to principal component analysis, specifically developed for use in categorical data [32]. The term Burt matrix refers to a cross-tabulation of all response categories. The subsequent analysis focused on the identification of sociodemographic determinants of participants’ knowledge about eye diseases. For this purpose, correct answers to the statements about eye diseases were summed up to a total score (maximum score 16 points) and sub-scores per disease (maximum score 4 points). Participants’ sub-scores were compared using the Friedman test. Associations between sample characteristics and test performance were investigated, using logistic regression models with the dichotomized sum score of correct responses by median as the dependent variable. Statistical analyses were performed with SPSS Statistics, version 27 (IBM Corporation, Armonk, NY), Winsteps, version 3.92.1 (Chicago, IL) and R, version 4.2.2 (Vienna, Austria). *P*-values < 0.05 were considered statistically significant.

## Results

A total of 1,008 participants completed the survey. Participants were on average 50 years old (age range: 18–86 years) and the majority did not report any visual difficulties. More than 90% of participants had undergone upper secondary or tertiary education (Table 1). More respondents had consulted a general practitioner over the previous 12 months (747 participants, 74.1%) than an ophthalmologist (398 participants, 39.5%). A small proportion of participants reported having been diagnosed with cataract, glaucoma, AMD or diabetic eye disease, respectively (6.9%, 4.3%, 4.9% and 4.4%).

**Table 1:** Sociodemograhic characteristics of the sample (*n* = 1,008)

	Mean ± SD or n (%)
Age, years	50 ± 16
Sex	
Female (%)	500 (49.6)
Male (%)	501 (49.7)
Diverse	3 (0.3)
Missing	4 (0.4)
Education	
Primary or lower secondary	41 (4.1)
Upper secondary^a^	446 (44.2)
Tertiary^b^	491 (48.7)
Other^c^	14 (1.4)
Missing	16 (1.6)
Employment	
Employed or self-employed	567 (56.3)
Retired	272 (27.0)
Student	43 (4.3)
Unemployed	49 (4.9)
Other	73 (7.2)
Missing	4 (0.4)
Marital status	
Single	314 (31.2)
Married / partner	541 (53.7)
Divorced	92 (9.1)
Widowed	50 (5.0)
Missing	11 (1.1)
Monthly household income	
< EUR 1,300	128 (12.7)
EUR 1,300 to < 1,700	114 (11.3)
EUR 1,700 to < 2,600	242 (24.0)
EUR 2,600 to < 3,600	155 (15.4)
EUR 3,600 to < 5,000	234 (23.2)
≥ EUR 5,000	94 (9.3)
Missing	41 (4.1)
EQ-5D-5L	
Value	0.84 ± 0.22
Mobility, problems (%)^d^	365 (36.2)
Self-care, problems (%)^d^	168 (16.7)
Usual activities, problems (%)^d^	330 (32.7)
Pain/discomfort, problems (%)^d^	586 (58.1)
Anxiety/depression, problems (%)^d^	442 (43.8)
General health	
Excellent (%)	53 (5.3)
Very good (%)	230 (22.8)
Good (%)	437 (43.4)
Fair (%)	247 (24.5)
Poor (%)	41 (4.1)
Visual difficulties	
No difficulties (%)	648 (64.3)
Some difficulties (%)	327 (32.4)
Large difficulties (%)	27 (2.7)
Cannot see (%)	6 (0.6)

^a^School education after age 16

^b^University degree or similar

^c^participants that reported no school education after age 16 and a University or similar degree

^d^Problems refer to the wording of the EQ-5D-5L response categories (Levels 2 to 5)

Out of sixteen statements about cataract, glaucoma, AMD and diabetic eye disease, participants correctly identified a median of 9 statements (range: 2 to 16 statements; Table 2). No responses were missing. The number of correct responses was significantly different between disease categories (*p* < 0.0001), with cataract scoring highest and AMD scoring lowest, while the categories glaucoma and diabetic eye disease ranked similarly (*p* = 0.743).

**Table 2:** Items included in the assessment of knowledge about eye diseases and proportion of correct responses

Category	Statement	Response correct (%)
Cataract	*4 items*	270 (26.8)^a^
C1	Risk factor age (true)	639 (63.4)
C2	Affects the optic nerve (false)	654 (64.9)
C3	Curable with surgical intervention (true)	631 (62.6)
C4	Curable with eye drops (false)	977 (96.9)
Glaucoma	*4 items*	89 (8.8)^a^
G1	Risk factor intraocular pressure (true)	624 (61.9)
G2	Risk factor family history (true)	271 (26.9)
G3	Curable with surgical intervention (false)	605 (60.0)
G4	Curable with eye drops (false)	919 (91.2)
AMD	*4 items*	13 (1.3)^a^
A1	Risk factor age (true)	609 (60.4)
A2	Curable with laser intervention (false)	538 (53.4)
A3	Risk reduction with diet and physical activity (true)	188 (18.7)
A4	Risk factor smoking (true)	137 (13.6)
Diabetic eye disease	*4 items*	189 (18.8)^a^
D1	Risk reduction with regular eye screenings (true)	635 (63.0)
D2	Blindness as a complication (true)	536 (53.2)
D3	Curable with laser intervention (false)	894 (88.7)
D4	Risk reduction with blood sugar management (true)	435 (43.2)

^a^Correct replies to all statements within category, *AMD* Age-related macular degeneration

### Internal consistency and dimensionality

**Fig. 1:**
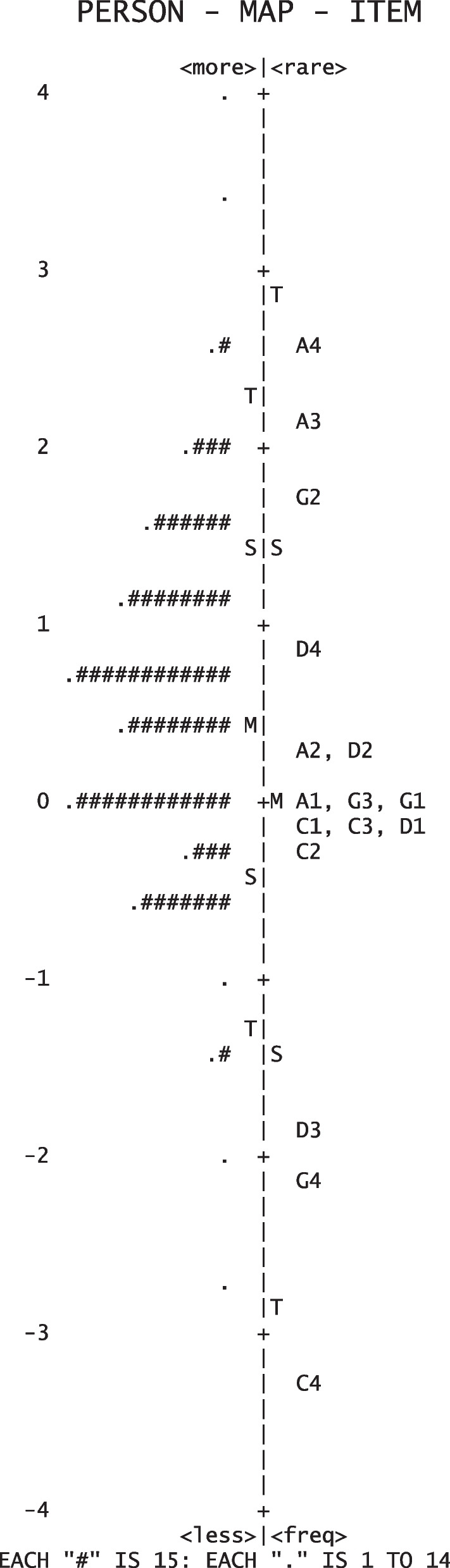
Person-item map derived from a Rasch model describing all 16 items on the knowledge about common eye diseases in Germany. Items on cataract (C), glaucoma (G), age-related macular degeneration (A) and diabetic eye disease (D) follow the descriptions from Table 2. The # and. symbols reflect groups of 15 and < 15 test takers at a defined knowledge level, respectively

The person reliability and person separation index in a dichotomous Rasch model from the responses were 0.46 and 0.92, respectively. Mean-square values ranged between 0.84 and 1.14 in the inlier-sensitive fit statistic and between 0.80 and 1.77 in the outlier-sensitive fit statistic. The person-item map indicated that the difficulty of the 16 items was well targeted to our population, with item A4 being most difficult and item C4 being least difficult (Fig. 1). One item (A3) was indicative of DIF for sex, with a higher probability of correct replies in female than in male participants (*p* = 0.0001, DIF contrast 0.69). The item was retained as the contrast threshold of 1.0 was not exceeded. Since the model requirements were not met and did not improve with any revisions, we decided not to use the Rasch model for further analysis.

**Fig. 2:**
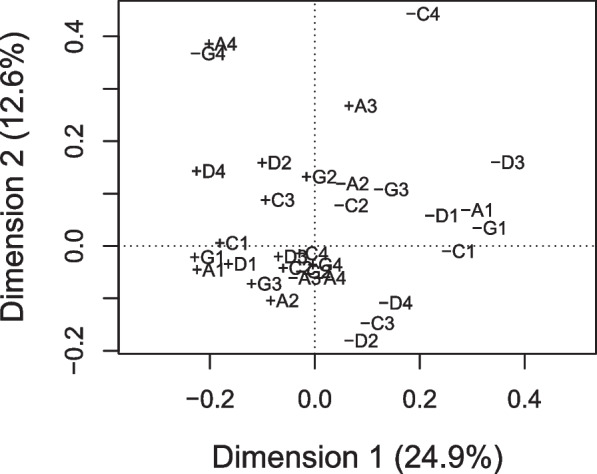
Biplot from multiple correspondence analysis based on correspondence analysis of the Burt matrix of correct ( +) and incorrect (-) responses to 16 items assessing knowledge about common eye diseases in Germany. Items on cataract (C), glaucoma (G), age-related macular degeneration (A) and diabetic eye disease (D) follow the descriptions from Table 2

In MCA, the first two dimensions explained 37.6% of the variance in the dataset (Fig. 2). Applying a rescaling algorithm that fit off-diagonal submatrices yielded 52.7% of the variance explained by the first two dimensions and a comparable biplot to Fig. 2 (data not shown). Most correct responses loaded negatively on the first dimension and all items loading positively on the second dimension included correct statements while no associations with the eye condition or the field of knowledge were qualitatively identified. As a result of MCA, all 16 items were used for further analysis.

### Determinants of knowledge

Five hundred twenty-four participants (52.0%) had nine correct responses or less (lower-knowledge group) and 484 participants (48.0%) had ten or more correct responses (higher-knowledge group). The median number of correct responses in these subgroups was 8 and 11, respectively. Based on this we dichotomized the dataset. Binary-logistic regression analysis revealed significant associations of being in the higher knowledge group with older age, retirement, reduced general health and consultations with general practitioners and ophthalmologists within the previous 12 months, while the level of education was not significantly associated (Table 3). These results remained overall consistent when looking at the best- and worst-performing participants of the sample by the highest- and lowest knowledge quartiles instead (Supplementary Table 1) and when excluding participants with self-reported cataract, glaucoma, AMD and diabetic eye disease (Supplementary Table 2).

**Table 3:** Factors associated with higher overall knowledge about common eye diseases in univariable binary-logistic regression models. Dependent variable: Result better or worse than the median (> versus ≤ 9 correct replies)

Independent variable	OR [95% CI]	*p*-value
**Age**	**1.014 [1.006;1.022]**	** < 0.0001**
Sex^a^ (female)	1.266 [0.988; 1.623]	0.062
Education	0.962 [0.845; 1.097]	0.565
**Employment**^b^ (retired)	**1.517 [1.134; 2.029]**	**0.005**
Marital status^c^ (no partner)	0.929 [0.724; 1.192]	0.563
Monthly household income	1.042 [0.960; 1.132]	0.328
EQ-5D-5L value	0.905 [0.522; 1.568]	0.721
**General health reduction**	**1.222 [1.067; 1.400]**	**0.004**
Visual difficulties	1.176 [0.948; 1.460]	0.141
**Consultation general practitioner**	**1.462 [1.256; 1.701]**	** < 0.0001**
**Consultation ophthalmologist**	**1.364 [1.200; 1.551]**	** < 0.0001**

Statistically significant assocations are marked in bold

^a^Considers male and female participants

^b^Considers categories employed/self-employed and retired

^c^Considers categories single/divoreced/windowed and married/partnership

In a single multivariable binary-logistic regression model, higher knowledge was significantly associated with both consultations with a general practitioner and an ophthalmologist over the past 12 months, when controlling for participants’ age, sex, employment status and general health (Table 4). On average, participants who had consulted a general practitioner or an ophthalmologist replied correctly to one of the statements more than participants who had not consulted these medical specialties, respectively. Analyses at an individual item level were consistent with these results (Supplementary Fig. 1).

**Table 4:** Factors associated with higher overall knowledge about common eye diseases in a multivariable binary-logistic regression model. Dependent variable: Result better or worse than the median (> versus ≤ 9 correct replies)

Independent variable	OR [95% CI]	*p*-value
Age	1.010 [0.998; 1.022]	0.117
Sex^a^ (female)	1.158 [0.875; 1.532]	0.305
Employment^b^ (retired)	1.107 [0.734; 1.671]	0.627
General health reduction	0.941 [0.789; 1.123]	0.502
**Consultation general practitioner**	**1.275 [1.058; 1.538]**	**0.011**
**Consultation ophthalmologist**	**1.256 [1.079; 1.462]**	**0.003**

Statistically significant associations are marked in bold

^a^Considers male and female participants

^b^Considers categories employed/self-employed and retired

## Discussion

The results of our study suggest that the knowledge of common age-related eye diseases in the adult German population is relatively poor, with more than one in three participants replying incorrectly to the majority of items. The knowledge assessment differed by disease: cataract-related items scored highest and AMD-related items scored lowest, indicating a relevant gap in eye health knowledge. Infrequent users of healthcare services as well as younger individuals, individuals in good general health and people in employment were less knowledgeable and could be relevant target groups for future eye health education interventions for the general population. Overall, our results emphasize the crucial role of healthcare personnel or contact with the healthcare system in improving SDOH and thus, health outcomes.

Our study confirms findings from other countries which demonstrated that AMD-related knowledge among the general population seems to be particularly poor, despite AMD being the most common cause of blindness and severe visual loss in all high-income countries [15–20, 33–35]. To date, no other study has assessed AMD-related knowledge in a demographically representative sample in Germany. Concerningly, less than 20% of our study participants knew common modifiable risk factors of AMD. In a previous study, we found that the majority of community dwelling elderly people were unaware of their AMD [36]. With an expected increase in AMD prevalence by 2040 [35, 37], public campaigns targeting this knowledge gap are urgently needed. This also holds true for diabetic eye disease, the most common global cause of blindness and vision loss in the working age population [38], for which more than a third of our study participants lacked knowledge about eye screening as an important prevention strategy. These knowledge gaps are supported by the results of previous studies [39–42], which is especially concerning given that early prevention strategies are highly effective in reducing the incidence of vision loss due to diabetic eye disease [43].

Much previous research focused on glaucoma [15–17, 19, 20, 22, 26, 44–50], where early detection is particularly important [51]. A 20-year old study in Germany demonstrated that glaucoma awareness was relatively high but participants struggled naming correct disease features and symptoms [44]. In our cohort, more than a third of respondents did not know that increased intraocular pressure was a risk factor for glaucoma and only one fourth knew that a positive family history increased the risk of developing glaucoma. This is particularly concerning since glaucoma is a relatively common condition and late presentation of glaucoma patients is associated with a poor prognosis [52, 53]. Anecdotally, glaucoma and cataract are often confused by the general public and 35% of respondents believed cataract directly affected the optic nerve. Overall, significantly more responses to cataract than glaucoma items were correct, despite early detection playing a crucial role in preventing irreversible glaucomatous visual field damage [54].

Lastly, the level of knowledge in our study was significantly associated with recent contact with the healthcare system (i.e. ophthalmologist or GP consultations), as well as older age, lower general health and employment status. A positive association between disease knowledge and age has been identified in previous studies which may be explained by the increased exposure to the healthcare system with older age. However, results in the literature are inconclusive [16, 18, 23]. Overall, more research is needed to better understand the determinants of eye health knowledge in infrequent users of the healthcare system and to tailor public awareness campaigns and education measures to these groups.

We developed a 16-item instrument (Supplementary Table 3) which covers knowledge about multiple ophthalmic diseases relevant in a population setting. Previous developments are mostly condition-specific (e.g., National Eye Health Education Program Eye-Q test, Auckland Glaucoma Knowledge Questionnaire, Diabetic Retinopathy Knowledge and Attitudes questionnaire [55–57]) and therefore not applicable for a study assessing knowledge across a spectrum of disorders. Interestingly, the results of a multiple correspondence analysis suggest that different knowledge domains (risk factors, prevention strategies, treatments, misconceptions across diseases) can be tested in a single knowledge assessment and support the internal consistency of the instrument. Its validity has been demonstrated by the qualitative development process and its construct validity is supported by the associations with sociodemographic factors that were associated with poorer health knowledge in the previous literature. Potential DIF in item A3 requires further investigation in future use scenarios of the instrument but could also be explained by different physical activity attitudes specific to our sample. The developed instrument could be used to assess the impact of healthcare education interventions in ophthalmology. However, further work is needed to refine the instrument.

The strengths of our study include the large sample size, which was demographically representative of the adult German population, performance-based assessment of knowledge instead of relying on self-evaluation, as well as use of modern psychometric methods. Limitations include content development based on literature and clinician input only, excluding patients. However, methods in the literature vary and it is arguable if patient involvement without involvement of the general public fully meets content validity requirements where standards are lacking. The assessment did not include disease awareness or other indicators of in-depth understanding of the disease. Our sample was not representative of the German population in terms of socioeconomics or geographic location, and the survey was conducted online, which may have led to selection bias, as indicated by the higher proportion of higher education backgrounds in our data compared to the general population. Our results could therefore underestimate the actual level of knowledge about age-related eye diseases in adult people in Germany. The knowledge instrument requires further reliability and validity assessment in the future before it can be applied in other settings, including an assessment of repeatability, concurrent validity and predictive validity. We included only few participants in the cognitive debriefings of our instrument and further qualitative work will be required to better understand how knowledge about common eye disease is best assessed for targeted health education interventions. Nevertheless, our study provides the most comprehensive assessment of eye health knowledge in the German general population.

## Conclusions

The knowledge of cataract, glaucoma, AMD and diabetic eye disease in Germany is relatively poor and health campaigns are required to educate the general public about eye health, most urgently AMD. Considering the expected increase in age-related eye disorders with asymptomatic early stages, education about risk factors and preventive measures merits a special focus.

### Supplementary Information

**Additional file 1: Supplementary Table 1.** Factors associated with the overall knowledge about common eye diseases in multiple, univariable binary-logistic regression analyses in the total sample. Dependent variable: Highest quartile (≥ 11 replies correct) versus lowest quartile (≤ 7 replies correct) of correct replies to all 16 items. **Supplementary Table 2.** Factors associated with the overall knowledge about common eye diseases in multiple, univariable binary-logistic regression analyses in a subgroup of 827 individuals with no reported cataract, glaucoma, age-related macular degeneration and diabetic eye disease. Dependent variable: Result better or worse than the median (> versus ≤ 9 correct replies). **Supplementary Table 3.** Knowledge assessment used in the study. **Supplementary Figure 1.** Factors associated with responses to single items in multivariable binary-logistic regression models. Odd’s ratios are provided where statistically significant. At an individual item level, age and consultations with a general practitioner as well as an ophthalmologist over the previous 12 months were significantly associated with participants’ knowledge about cataract, glaucoma, AMD and diabetic eye disease. In addition, sex was significantly associated with knowledge about glaucoma, AMD and diabetic eye disease. Interestingly, item A4 (AMD risk factor smoking) was inversely associated with recent general practitioner consultations, and the items C4 and G4 (eye drops as curative therapy in cataract and glaucoma) were inversely associated with ophthalmologist consultations.

## Data Availability

The data that support the findings of this study are accessible via University Hospital Bonn, Department of Ophthalmology, via the corresponding author on reasonable request.

## Abbreviations

AMD: Age-related macular degeneration
MCA: Multiple correspondence analysis
SDOH: Social determinants of health

## References

[1] Hood CM, Gennuso KP, Swain GR, Catlin BB (2016). County health rankings: relationships between determinant factors and health outcomes. Am J Prev Med.

[2] Capó H, Edmond JC, Alabiad CR, Ross AG, Williams BK, Briceño CA (2022). The importance of health literacy in addressing eye health and eye care disparities. Ophthalmology.

[3] Williams AM, Sahel J-A (2022). Addressing social determinants of vision health. Ophthalmol Ther.

[4] Nutbeam D (2000). Health literacy as a public health goal: a challenge for contemporary health education and communication strategies into the 21st century. Health Promot Int.

[5] Mogford E, Gould L, Devoght A (2011). Teaching critical health literacy in the US as a means to action on the social determinants of health. Health Promot Int.

[6] Elam AR, Tseng VL, Rodriguez TM, Mike EV, Warren AK, Coleman AL (2022). Disparities in vision health and eye care. Ophthalmology.

[7] Berkman ND, Sheridan SL, Donahue KE, Halpern DJ, Crotty K (2011). Low health literacy and health outcomes: an updated systematic review. Ann Intern Med.

[8] Muir KW, Christensen L, Bosworth HB (2013). Health literacy and glaucoma. Curr Opin Ophthalmol.

[9] Zoega GM, Gunnarsdóttir T, Björnsdóttir S, Hreietharsson AB, Viggósson G, Stefánsson E (2005). Screening compliance and visual outcome in diabetes. Acta Ophthalmol Scand.

[10] Schwennesen N, Barghadouch A, Olesen K (2019). Health Literacy and self-care among visually impaired people with type 1 diabetes in Denmark. Chronic Illn.

[11] Lian J, McGhee SM, Gangwani RA, Lam CLK, Yap MKH, Wong DSH (2018). Awareness of diabetic retinopathy and its association with attendance for systematic screening at the public primary care setting: a cross-sectional study in Hong Kong. BMJ Open.

[12] Drinkwater JJ, Chen FK, Davis WA, Davis TME (2021). Knowledge of ocular complications of diabetes in community-based people with type 2 diabetes: The fremantle diabetes study II. Prim Care Diabetes.

[13] Klein BEK, Klein R. Projected prevalences of age-related eye diseases. Invest Ophthalmol Vis Sci. 2013;54:ORSF14–7. 10.1167/iovs.13-12782. 10.1167/iovs.13-12782 PMC3864373 24335061

[14] GBD 2019 Blindness and Vision Impairment Collaborators. Causes of blindness and vision impairment in 2020 and trends over 30 years, and prevalence of avoidable blindness in relation to VISION 2020: the Right to Sight: an analysis for the Global Burden of Disease Study. Lancet Glob Health. 2021;9:e144-e160. 10.1016/S2214-109X(20)30489-7. 10.1016/S2214-109X(20)30489-7 PMC7820391 33275949

[15] Wong PW, Lau JK, Choy BN, Shih KC, Ng AL, Chan JC, Wong IY (2020). Epidemiological factors associated with health knowledge of three common eye diseases: A community-based pilot survey in Hong Kong. SAGE Open Med.

[16] Livingston PM, McCarty CA, Taylor HR (1998). Knowledge, attitudes, and self care practices associated with age related eye disease in Australia. Br J Ophthalmol.

[17] Islam FMA, Chakrabarti R, Islam SZ, Finger RP, Critchley C (2015). Factors associated with awareness, attitudes and practices regarding common eye diseases in the general population in a rural district in Bangladesh: The Bangladesh Population-based Diabetes and Eye Study (BPDES). PLoS ONE.

[18] Noertjojo K, Maberley D, Bassett K, Courtright P (2006). Awareness of eye diseases and risk factors: identifying needs for health education and promotion in Canada. Can J Ophthalmol.

[19] Lau JTF, Lee V, Fan D, Lau M, Michon J (2002). Knowledge about cataract, glaucoma, and age related macular degeneration in the Hong Kong Chinese population. Br J Ophthalmol.

[20] Attebo K, Mitchell P, Cumming R, Smith W (1997). Knowledge and beliefs about common eye diseases. Aust N Z J Ophthalmol.

[21] Haddad MF, Bakkar MM, Abdo N (2017). Public awareness of common eye diseases in Jordan. BMC Ophthalmol.

[22] Thapa SS, Berg RVD, Khanal S, Paudyal I, Pandey P, Maharjan N (2011). Prevalence of visual impairment, cataract surgery and awareness of cataract and glaucoma in Bhaktapur district of Nepal: the Bhaktapur Glaucoma Study. BMC Ophthalmol.

[23] Thapa R, Bajimaya S, Paudyal G, Khanal S, Tan S, Thapa SS, van Rens G (2015). Population awareness of diabetic eye disease and age related macular degeneration in Nepal: the Bhaktapur Retina Study. BMC Ophthalmol.

[24] Michielutte R, Diseker RA, Stafford CL, Carr P (1984). Knowledge of diabetes and glaucoma in a rural North Carolina community. J Community Health.

[25] Alammar AA, Alabdulkareem AM, Abu-Amara AB, Kalantan H (2021). Assessment of the levels of knowledge regarding cataract and glaucoma in Saudi Arabia and measurement of the ability to differentiate between the two. Cureus.

[26] Zhao M, Gillani AH, Amirul Islam FM, Ji W, Hayat K, Li Z (2019). Factors associated with knowledge, attitude and practices of common eye diseases in general population: A multicenter cross-sectional study from Pakistan. Int J Environ Res Public Health.

[27] Krejcie RV, Morgan DW (1970). Determining sample size for research activities. Educ Psychol Measur.

[28] Federal Statistical Office (Destatis). Genesis-Online. 18/10/2022.

[29] van Hout B, Janssen MF, Feng Y-S, Kohlmann T, Busschbach J, Golicki D (2012). Interim scoring for the EQ-5D-5L: mapping the EQ-5D-5L to EQ-5D-3L value sets. Value Health.

[30] Benjamin D Wright, Magdalena M C Mok. An Overview of the Family of Rasch Measurement Models.

[31] Pesudovs K, Burr JM, Harley C, Elliott DB (2007). The development, assessment, and selection of questionnaires. Optom Vis Sci.

[32] Abdi H, Valentin D. Multiple Correspondence Analysis. 2455 Teller Road, Thousand Oaks California 91320 United States of America: Sage Publications, Inc; 2007.

[33] Sanjay S, Neo HY, Sangtam T, Ku JY, Chau SYM, Rostihar AK, Au Eong K-G (2009). Survey on the knowledge of age-related macular degeneration and its risk factors among Singapore residents. Clin Exp Ophthalmol.

[34] Rosenthal B, Thompson B (2003). Awareness of age-related macular degeneration in adults: the results of a large-scale international survey. Optometry.

[35] Wong WL, Su X, Li X, Cheung CMG, Klein R, Cheng C-Y, Wong TY (2014). Global prevalence of age-related macular degeneration and disease burden projection for 2020 and 2040: a systematic review and meta-analysis. Lancet Glob Health.

[36] Heinemann M, Welker SG, Li JQ, Wintergerst MWM, Turski GN, Turski CA (2019). Awareness of age-related macular degeneration in community-dwelling elderly persons in Germany. Ophthalmic Epidemiol.

[37] Li JQ, Welchowski T, Schmid M, Mauschitz MM, Holz FG, Finger RP (2020). Prevalence and incidence of age-related macular degeneration in Europe: a systematic review and meta-analysis. Br J Ophthalmol.

[38] Teo ZL, Tham Y-C, Yu M, Chee ML, Rim TH, Cheung N (2021). Global Prevalence of diabetic retinopathy and projection of burden through 2045: systematic review and meta-analysis. Ophthalmology.

[39] Al-Yahya A, Alsulaiman A, Almizel A, Barri A, Al AF (2020). Knowledge, Attitude, and Practices (KAP) of diabetics towards diabetes and diabetic retinopathy in Riyadh Saudi Arabia: Cross-Sectional Study. Clin Ophthalmol.

[40] Cetin EN, Zencir M, Fenkçi S, Akın F, Yıldırım C (2013). Assessment of awareness of diabetic retinopathy and utilization of eye care services among Turkish diabetic patients. Prim Care Diabetes.

[41] Lee Y-H (2018). Socioeconomic differences among community-dwelling diabetic adults screened for diabetic retinopathy and nephropathy: The 2015 Korean Community Health Survey. PLoS ONE.

[42] Dandona R, Dandona L, John RK, McCarty CA, Rao GN (2001). Awareness of eye diseases in an urban population in southern India. Bull World Health Organ.

[43] Marozas LM, Fort PE (2014). Diabetic retinopathy-update on prevention techniques, present therapies, and new leads. US Ophthalmic Rev.

[44] Pfeiffer N, Krieglstein GK, Wellek S (2002). Knowledge about glaucoma in the unselected population: a German survey. J Glaucoma.

[45] Baker H, Cousens SN, Murdoch IE (2010). Poor public health knowledge about glaucoma: fact or fiction?. Eye (Lond).

[46] Mansouri K, Orgül S, Meier-Gibbons F, Mermoud A (2006). Awareness about glaucoma and related eye health attitudes in Switzerland: a survey of the general public. Ophthalmologica.

[47] Sathyamangalam RV, Paul PG, George R, Baskaran M, Hemamalini A, Madan RV (2009). Determinants of glaucoma awareness and knowledge in urban Chennai. Indian J Ophthalmol.

[48] Rewri P, Kakkar M (2014). Awareness, knowledge, and practice: a survey of glaucoma in north Indian rural residents. Indian J Ophthalmol.

[49] Alemu DS, Gudeta AD, Gebreselassie KL (2017). Awareness and knowledge of glaucoma and associated factors among adults: a cross sectional study in Gondar Town. Northwest Ethiopia BMC Ophthalmol.

[50] Yenegeta Z, Tsega A, Addis Y, Admassu F (2020). Knowledge of glaucoma and associated factors among adults in Gish Abay town. Northwest Ethiopia BMC Ophthalmol.

[51] Tatham AJ, Weinreb RN, Medeiros FA (2014). Strategies for improving early detection of glaucoma: the combined structure-function index. Clin Ophthalmol.

[52] Wilson R, Walker AM, Dueker DK, Crick RP (1982). Risk factors for rate of progression of glaucomatous visual field loss: a computer-based analysis. Arch Ophthalmol.

[53] Fraser S, Bunce C, Wormald R, Brunner E (2001). Deprivation and late presentation of glaucoma: case-control study. BMJ.

[54] Allison K, Patel D, Alabi O (2020). Epidemiology of glaucoma: the past, present, and predictions for the future. Cureus.

[55] Peralta E, Muir KW, Rosdahl JA (2018). Systematic review of knowledge assessments for glaucoma patients. Semin Ophthalmol.

[56] Skalicky SE, D'Mellow G, House P, Fenwick E (2018). Glaucoma Australia educational impact study: a randomized short-term clinical trial evaluating the association between glaucoma education and patient knowledge, anxiety and treatment satisfaction. Clin Exp Ophthalmol.

[57] Fenwick EK, Man REK, Gan ATL, Aravindhan A, Tey CS, Soon HJT (2020). Validation of a new diabetic retinopathy knowledge and attitudes questionnaire in people with diabetic retinopathy and diabetic macular edema. Transl Vis Sci Technol.

