# Supplementary material for: Knowledge about age-related eye diseases in the general population in Germany
Source: BMC Public Health. 2024 Feb 8;24:409. doi: 10.1186/s12889-024-17889-0 (PMC10851458; doi:10.1186/s12889-024-17889-0)
Supplement: Supplementary file 1 — Additional file 1: Supplementary Table 1. Factors associated with the overall knowledge about common eye diseases in multiple, univariable binary-logistic regression analyses in the total sample. Dependent variable: Highest quartile (≥ 11 replies correct) versus lowest quartile (≤ 7 replies correct) of correct replies to all 16 items. Supplementary Table 2. Factors associated with the overall knowledge about common eye diseases in multiple, univariable binary-logistic regression analyses in a subgroup of 827 individuals with no reported cataract, glaucoma, age-related macular degeneration and diabetic eye disease. Dependent variable: Result better or worse than the median (> versus ≤ 9 correct replies). Supplementary Table 3. Knowledge assessment used in the study. Supplementary Figure 1. Factors associated with responses to single items in multivariable binary-logistic regression models. Odd’s ratios are provided where statistically significant. At an individual item level, age and consultations with a general practitioner as well as an ophthalmologist over the previous 12 months were significantly associated with participants’ knowledge about cataract, glaucoma, AMD and diabetic eye disease. In addition, sex was significantly associated with knowledge about glaucoma, AMD and diabetic eye disease. Interestingly, item A4 (AMD risk factor smoking) was inversely associated with recent general practitioner consultations, and the items C4 and G4 (eye drops as curative therapy in cataract and glaucoma) were inversely associated with ophthalmologist consultations. [file 12889_2024_17889_MOESM1_ESM.docx]

**SUPPLEMENT**

| **Supplementary Table 1.** Factors associated with the overall knowledge about common eye diseases in multiple, univariable binary-logistic regression analyses in the total sample. Dependent variable: Highest quartile (≥ 11 replies correct) versus lowest quartile (≤ 7 replies correct) of correct replies to all 16 items | | |
| --- | --- | --- |
| **Independent variable** | **OR [95% CI]** | **p-value** |
| **Age** | **1.021 [1.009;1.032]** | **<0.0001** |
| Sex^*^ (female) | 1.135 [0.796; 1.620] | 0.484 |
| Education | 0.941 [0.780; 1.135] | 0.532 |
| **Employment^#^** (self-/employed) | **1.651 [1.082; 2.519]** | **0.020** |
| Marital status^✝^ | 0.954 [0.667; 1.366] | 0.799 |
| Monthly household income | 1.058 [0.939; 1.192] | 0.355 |
| EQ-5D-5L value | 1.232 [0.565; 2.687] | 0.600 |
| **General health reduction** | **1.417 [1.158; 1.735]** | **0.001** |
| Visual difficulties | 1.020 [0.748; 1.391] | 0.898 |
| **Consultation general practitioner** | **1.688 [1.370; 2.082]** | **<0.0001** |
| **Consultation ophthalmologist** | **1.543 [1.277; 1.864]** | **<0.0001** |
| ^*^ Considers male and female participants ^#^ Considers categories employed/self-employed and retired ^✝^ Considers categories single/divoreced/windowed and married/partnership Statistically significant assocations are marked in bold | | |

| **Supplementary Table 2.** Factors associated with the overall knowledge about common eye diseases in multiple, univariable binary-logistic regression analyses in a subgroup of 827 individuals with no reported cataract, glaucoma, age-related macular degeneration and diabetic eye disease. Dependent variable: Result better or worse than the median (> versus ≤ 9 correct replies) | | |
| --- | --- | --- |
| **Independent variable** | **OR [95% CI]** | **p-value** |
| **Age** | **1.012 [1.003;1.020]** | **0.009** |
| **Sex^*^** (female) | **1.319 [1.003; 1.736]** | **0.048** |
| Education | 0.994 [0.860; 1.149] | 0.940 |
| **Employment^#^** (self-/employed) | **1.393 [1.001; 1.939]** | **0.049** |
| Marital status^✝^ | 0.916 [0.696; 1.205] | 0.529 |
| Monthly household income | 1.049 [0.957; 1.149] | 0.307 |
| EQ-5D-5L value | 0.633 [0.316; 1.270] | 0.198 |
| **General health reduction** | **1.168 [1.003; 1.359]** | **0.045** |
| Visual difficulties | 1.145 [0.892; 1.470] | 0.289 |
| **Consultation general practitioner** | **1.390 [1.180; 1.637]** | **<0.0001** |
| **Consultation ophthalmologist** | **1.274 [1.099; 1.476]** | **0.001** |
| ^*^ Considers male and female participants ^#^ Considers categories employed/self-employed and retired ^✝^ Considers categories single/divoreced/windowed and married/partnership Statistically significant assocations are marked in bold | | |

| **Supplementary Table 3.** Knowledge assessment used in the study | |
| --- | --- |
| **Language** | **Assessment** |
| German^*^ | Die folgenden, abschließenden vier Fragen beschäftigten sich mit Ihrem Wissen zur Augengesundheit. Bitte beantworten Sie die Fragen nach bestem Wissen und ohne zusätzliche Informationsquellen (Internet, Bücher) zu nutzen.  Welche Aussagen zum grauen Star sind richtig?  (mehrfache Antworten möglich)  Der graue Star tritt häufig bei älteren Menschen auf.  Der graue Star ist eine Erkrankung des Sehnerven.  Der graue Star ist durch eine Operation heilbar.  Der graue Star ist durch Augentropfen heilbar.  Welche Aussagen zum grünen Star / Glaukom sind richtig?  (mehrfache Antworten möglich)  Der grüne Star ist häufig mit einem erhöhten Augeninnendruck verbunden.  Der grüne Star tritt häufiger auf, wenn man erkrankte Verwandte hat.  Der grüne Star ist durch eine Operation heilbar.  Der grüne Star ist durch Augentropfen heilbar.  Welche Aussagen zur altersabhängigen Makuladegeneration (AMD) sind richtig?  (mehrfache Antworten möglich)  Die altersabhängige Makuladegeneration (AMD) ist eine häufige Erkrankung bei älteren Menschen.  Die altersabhängige Makuladegeneration (AMD) ist durch einen Lasereingriff heilbar.  Der altersabhängigen Makuladegeneration (AMD) kann man durch eine gesunde Ernährung und Sport vorbeugen.  Die altersabhängige Makuladegeneration (AMD) tritt häufig bei Rauchern auf.  Welche Aussagen zur Augenerkrankung durch Diabetes sind richtig?  (mehrfache Antworten möglich)  Menschen mit Diabetes müssen regelmäßige Vorsorgeuntersuchungen beim Augenarzt machen lassen.  Eine Augenerkrankung durch Diabetes kann zur Erblindung führen.  Eine Augenerkrankung durch Diabetes ist durch einen Lasereingriff heilbar.  Einer Augenerkrankung durch Diabetes kann man durch eine dauerhaft gute Blutzuckereinstellung vorbeugen. |
| English^*^ | The following, concluding four questions will test your knowledge of eye health. Please answer the questions to the best of your knowledge and without using additional information sources (internet, books).  Which statements about cataracts are correct?  (multiple answers possible)  Cataracts are common in older people.  Cataracts are a condition of the optic nerve.  Cataracts are curable with surgery.  Cataracts are curable with eye drops.  Which statements about glaucoma are correct?  (multiple answers possible)  Glaucoma is often associated with increased intraocular pressure.  Glaucoma is more common in people with family members affected.  Glaucoma is curable with surgery.  Glaucoma is curable with eye drops.  Which statements about age-related macular degeneration (AMD) are correct?  (multiple answers possible)  Age-related macular degeneration (AMD) is a common disease in older people.  Age-related macular degeneration (AMD) is curable with laser surgery.  Age-related macular degeneration (AMD) can be prevented by a healthy diet and exercise.  Age-related macular degeneration (AMD) is more common in smokers.  Which statements about diabetes eye disease are correct?  (multiple answers possible)  People with diabetes need to have regular eye exams at the eye doctor.  Eye disease from diabetes can lead to blindness.  Eye disease from diabetes is curable with laser surgery.  Eye disease from diabetes can be prevented by maintaining good blood sugar control. |
| ^*^The questionnaire was administered in German, the English translation is provided for the international readership of the manuscript but did not follow translation guidelines | |


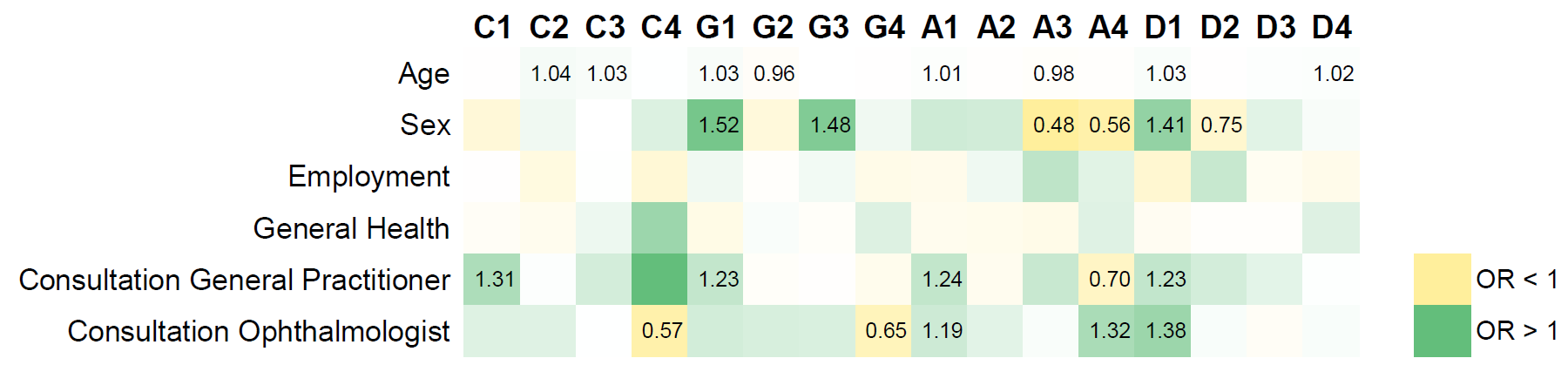


**Supplementary Figure 1.** Factors associated with responses to single items in
multivariable binary-logistic regression models. Odd’s ratios are provided where statistically significant. At an individual item level, age and consultations with a general practitioner as well as an ophthalmologist over the previous 12 months were significantly associated with participants’ knowledge about cataract, glaucoma, AMD and diabetic eye disease. In addition, sex was significantly associated with knowledge about glaucoma, AMD and diabetic eye disease. Interestingly, item A4 (AMD risk factor smoking) was inversely associated with recent general practitioner consultations, and the items C4 and G4 (eye drops as curative therapy in cataract and glaucoma) were inversely associated with ophthalmologist consultations
